# The efficiency of botulinum toxin type A for the treatment of masseter muscle pain in patients with temporomandibular joint dysfunction and tension-type headache

**DOI:** 10.1186/s10194-016-0621-1

**Published:** 2016-03-24

**Authors:** Malgorzata Pihut, Ewa Ferendiuk, Michal Szewczyk, Katarzyna Kasprzyk, Mieszko Wieckiewicz

**Affiliations:** Department of Dental Prosthetics, Jagiellonian University in Krakow, College of Medicine, Institute of Dentistry, 4 Montelupich St., 31-155 Krakow, Poland; Department of Neurology, Jagiellonian University in Krakow, College of Medicine, 3 Botaniczna St., 31-503 Krakow, Poland; Department of Prosthetic Dentistry, Faculty of Dentistry, Wroclaw Medical University, 26 Krakowska St., 50-425 Wroclaw, Poland

**Keywords:** Botulinum toxin, Masseter muscle pain, Temporomandibular joint dysfunction, Tension-type headache

## Abstract

**Background:**

Temporomandibular joint dysfunction are often accompanied by symptoms of headache such as tension-type headache which is the most frequent spontaneous primary headache. Masseter muscle pain is commonly reported in this group. The purpose of the study was to assess the efficiency of intramuscular botulinum toxin type A injections for treating masseter muscle pain in patients with temporomandibular joint dysfunction and tension-type headache.

**Methods:**

This prospective outcome study consisted of 42 subjects of both genders aged 19–48 years diagnosed with masseter muscle pain related to temporomandibular joint dysfunction and tension-type headache. The subjects were treated by the intramuscular injection of 21 U (mice units) of botulinum toxin type A (Botox, Allergan) in the area of the greatest cross-section surface of both masseter bellies. Pain intensity was evaluated using visual analogue scale (VAS) and verbal numerical rating scale (VNRS) 1 week before the treatment and 24 weeks after the treatment. The obtained data were analyzed using the Wilcoxon matched pairs test (*p* ≤ 0,005).

**Results:**

The results of this study showed a decrease in the number of referred pain episodes including a decrease in pain in the temporal region bilaterally, a reduction of analgesic drugs intake as well as a decrease in reported values of VAS and VNRS after injections (*p* = 0,000).

**Conclusions:**

The intramuscular botulinum toxin type A injections have been an efficient method of treatment for masseter muscle pain in patients with temporomandibular joint dysfunction and tension-type headache.

## Background

Symptoms characteristic for temporomandibular joint dysfunction (TMJD) such as masticatory muscles pain, temporomandibular joint pain, derangements of the condyle-disc complex and deviations of mandible movements are often accompanied by symptoms that are not directly related to the functioning of the temporomandibular joint [1–8]. Such signs include otologic symptoms (ear pain, tinnitus, vertigo), neurovascular headaches and tension-type headaches (TTH) [9–13]. TTH are the most frequent spontaneous primary headaches. They are observed more frequently in women, and occurred in all age groups. It should be emphasized that in most cases the TTH affect middle-aged patients. This kind of headache was also observed in approximately 5–7 % of students aged 5–15 years. The American Dental Association stated that more than 15 % of American adults suffer from chronic headache pain [11–16].

Diagnostics of TTHs is based on the data collected in a screening history consisted short questions which let to analyze the background of the pain and the factors responsible for pain origin. Specialized neuroimaging modalities (magnetic resonance, angiography, positron emission tomography) are used less frequently. The results of additional tests provide to exclude other causes of the TTH, especially migraine headache, aseptic meningitis, neuroborreliosis or pseudotumor cerebri [11, 17–18]. The following disorders should also be taken into consideration in differential diagnosis: hemicrania continua, spinal cord injury, central nervous system disorders and depression. Sinus pain, medication-induced headache and intracranial hypertension may also be important. TTH may be caused by psychoemotional factors, chronic stress, fatigue, sleep disorders and severe dehydration [17–22].

Bilateral, constant, dull ache of mild to moderate intensity without preexisting aura, vomiting, nausea is characteristic of TTH. Although TTH are not as widely recognized as migraine headaches, they constitute an important and frequent clinical problem, as they exert negative impact on the patients’ quality of life. Tension-type headache is affecting the temporal and occipital region. The patient may also report the feeling of squeezing within the head. In the beginning the headache is not intensive. Later it could intensify and gain the same level of pain as the migraine headache. Moreover, the frequency at which TTH occurs is important for diagnosis: it lasts at least 30 min daily, occurs on 15 days in a month, affects the patient for more than 3 months, and is detected in all age groups, but it affects middle-aged people most frequently. TTH are frequently accompanied by sleep disorders, chronic fatigue syndrome, noise hypersensitivity and appetite loss [13, 15, 22]. Risk factors include: gender (women are affected more frequently), hormone changes, emotional stress, depression, anxiety and genetic factors. Moreover, TTH could be a result of head and neck injury, bruxism, and psychoactive and analgesic drug intake. According to the latest edition of the International Classification of Headache Disorders (ICHD-3 beta) prepared by the International Headache Society, TTH can be classified into: infrequent tension-type headaches, frequent tension-type headaches, chronic tension-type headaches, and probable tension-type headaches. Tension-type headaches accompany temporomandibular joint dysfunction with varying frequency because head and neck muscles remain in close anatomical and physiological relationship [22–26].

The aim of this prospective outcome study was to assess the efficiency of intramuscular botulinum toxin type A (BTXA) injections in a case of masseter muscle pain in patients with temporomandibular joint dysfunction and tension-type headache.

## Methods

This is a prospective outcome study which consisted of 42 subjects of both genders, aged 19–48 years (mean age was 30) with masseter muscle pain related to temporomandibular joint dysfunction and tension-type headache. Patients were recruited from the Department of Dental Prosthetics at the Jagiellonian University in Krakow during the years 2009–2014 and were included in the study if they met the following criteria: (1) presence of TMJD which include unilateral or bilateral disc displacement with or without reduction, arthralgia, degenerative joint disease, subluxation, (2) masseter muscle pain, (3) increased masticatory muscles tension, (4) TTH, (5) absence of previous neurological treatment due to headache and a head injury within 6 years and (6) patient consent to be involved in the study. The rest of patients were excluded because of general (known hypersensitivity to BTXA, myasthenia gravis, Eaton-Lambert syndrome, pregnancy or lactation and taking aminoglycosides or curare-like compounds) and/or local (infection at the proposed site of injection) contraindications for intramuscular botulinum toxin type A injections as well as absence of consent to be involved in the study.

Clinical assessment of temporomandibular joints and masticatory muscles was performed by one experienced and self-trained examiner according to the RDC/TMD recommendations [27, 28]. Further diagnostics was based on survey and clinical examination according to the International Headache Society guidelines performed by experienced physician in the Department of Neurology at the Jagiellonian University in Krakow [29]. According to the performed examination there was no indications for neuroimaging examination due to the headache in the study group. The study protocol has been approved by the Bioethical Committee of the Jagiellonian University in Krakow No: KBET/96/B/2007.

The data collected during the survey have been important for the purpose of the study: localization of the headache, pain duration, and factors responsible for pain origin. The patients were also asked whether the pain was constant, episodic, recurrent, referred, and whether it was felt as dull, sharp, burning or stinging. It was important whether the patient reported that the pain was squeezing the head as well as previous treatment due to the headache. Authors paid close attention to symptoms that accompanied pain, such as: sleep disorders, chronic fatigue, noise hypersensitivity, and pain referral within the face or other areas of the head. An important aspect was the necessity for analgesic drug administration.

After patient enrollment the treatment of masseter muscle pain consisted of intramuscular injection of 21 U (mice units) of type A botulinum toxin (Botox, Allergan), in the area of the greatest cross-section surface of both masseter bellies.

Clinical algesimetry, e.g. the evaluation of pain intensity with the use of various scales is not devoid of subjective influence. However, it is the currently indicated method of measuring pain intensity at following appointments. For the purpose of the study two scales were applied by the authors: VAS (Visual Analogue Scale) and VNRS (Verbal Numerical Rating Scale). VAS is a psychometric response scale which can be used in questionnaires [30]. It is a measurement instrument for characteristics or attitudes that cannot be directly measured. Participants specify their level of pain intensity to a statement by indicating a position along a continuous line between two end-points (0–10). VNRS comprises assessment that is based on a numerical 10–point scale (0–10) in combination with a color-coded scale in which the increase in the score is accompanied by the increase in color intensity indicated on the scale. Mean intensity of pain was evaluated by subjects 1 week before the injection (examination I) and 24 weeks after the injection because of potential absence of BTXA activity (examination II).

The results were analyzed using the Wilcoxon matched pairs test, with statistical significance at *p* ≤ 0,005. The software used in the statistical analysis was STATISTICA version 8 (StatSoft Inc., Tulsa, Oklahoma, USA).

## Results

The most frequently reported complaints included: spontaneous masseter muscle pain and/or temporomandibular joint pain, clicking in the temporomandibular joint during mandible movements, impaired mastication and tension-type headaches in the anterior temporal region, medial temporal region and/or occipital region. The pain was dull, squeezing, or crushing, rarely encircling the head, and it lasted for minimum four hours daily and had been present for at least 4 months. It was unpleasant for the patients but it did not interfere with their everyday quality of life. Medication- and injury-induced migraine headache was excluded.

**Fig. 1:**
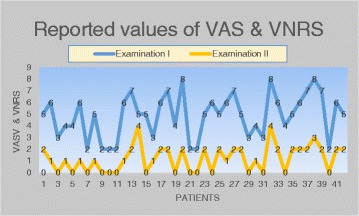
The values of VAS & VNRS reported in examination I and II

Table 1 presents the clinical parameters of reported headaches which have been diagnosed in the study group during examination I and II such as characteristics of pain, pain duration, accompanying symptoms of pain, referral of pain and applied analgesic drugs. The collected data have shown the valid decrease in the number of each parameter. The headache intensity which have been assessed using VAS & VNRS are presented in Fig. 1. The statistical analysis is presented in Table 2 and showed a significant decrease of reported VAS & VNRS values in examination II (*p* = 0,00000).

**Table 1:** The number of reported pain parameters collected in examination I and II

Tension-type headache			Examination I	Examination II
1. Characteristics of pain	Spontaneous headache	Unilateral	13	7
		Bilateral	29	20
	Provoked headache	Unilateral	8	3
		Bilateral	13	9
	Dull		13	5
	Squeezing		9	4
	Crushing		10	3
	Encircling		6	1
	Throbbing		4	2
2. Pain duration	Daily/h		4–6	2–3
	Weekly/days		5	3
3. Accompanying symptoms of pain	Sleep disorders		19	5
	Chronic fatigue		28	4
	Noise hypersensitivity		5	1
	Appetite loss		6	1
4. Referral of pain	Within the face		16	5
	Within the head		9	1
5. Applied analgesic drugs	Non-steroidal anti-inflammatory drugs		18	2
	Paracetamol		8	1

**Table 2:** The results of statistical analysis concerning reported values of VAS & VNRS

	Examination I	Examination II
Average ± SD	4,86 ± 1,84	1,21 ± 1,12
Median	5	1
Min - Max	2–8	0–4
Wilcoxon matched pairs test	*p* = 0,00000	

It is important that mean value of headache intensity during examination I was 4,86 points (maximal value 8), while the result of examination II was only 1,21 (maximal value 4). The difference between them was statistically significant because *p* = 0,000. The comparison of the examination I and II data have shown a positive changes in tension–type headache intensity. The differences mostly included: a decrease in the number of subjects with bilateral pain in the temporal region and lower number of referred pain episodes, as well as a reduction in the amount of analgesic drugs intake.

## Discussion

Independently from the results of various studies, the relationship between tension-type headaches and temporomandibular joint dysfunction can be confirmed by the decrease in headache intensity observed after the management of temporomandibular joint dysfunction. It is apparent particularly in cases in which no significant improvement in the patient’s well-being is observed after conventional neurological treatment or in which a quick recurrence of the symptoms occurs if temporomandibular joint dysfunction treatment is not initiated [5, 9, 10, 13, 17].

The aim of the population-based cross-sectional study conducted by Goncalves et al. was to determine the coexistence of TTH and TMJD in adult patients [31]. The results of their study indicate that such coexistence is observed frequently and that those two entities should be discussed together. The use of intramuscular BTXA injections within the masseter muscle led to positive alterations in pain intensity and the nature of complaints related with tension-type headache. Botox is, however, routinely deposited for neurological purposes within the temporal, occipital, and quadriceps muscles [28, 32–37].

The decrease in reported the headache following pharmacotherapy of temporomandibular joint dysfunction suggests that tension-type headaches are in many cases related to excessive and long-lasting tension within the muscles of the temporomandibular joint, which remains closely interrelated within the head and neck. After the injections, the character of tension-type headache changed and its intensity decreased. Moreover, a decrease in the daily number of hours and monthly number of days during which tension-type headache affected the patient, was observed [34, 37–40].

Numerous authors in contemporary literature underline the role played by stress in the development of TTH. At the same time, for several years authors of various studies concerning etiological factors of TMJD have been underlining that stress is an increasingly important etiological factor in its development [41]. Also, the results of studies by Yancey et al., which showed that psychorelaxation treatment and behavioral techniques were effective in treating this pathology, indicate that psychogenic factors play an important role in the development of tension-type headaches [42].

Jackson et al. have shown in the meta-analysis concerning the use of BTXA in the prophylaxis of migraine and tension-type headaches that the discussed drug had a positive effect in both disorders [36]. According to them, however, one should pay attention to the possibility of complications related with the application method of the drug. Singh and Sahota stressed that Botox plays a key role in the treatment of chronic headaches, independently from essential education concerning hygiene and lifestyle modification [43].

The results of in vivo and in vitro research performed by Ashkenazi and Blumenfeld have shown that botulinum toxin type A is effective in reducing tension-type headache intensity [44]. The study has shown that this drug is effective, safe, and well tolerated in the treatment of headache. The drug is administered every 12 weeks, which is convenient for some patients when compared with taking analgesic drugs every day [45]. Mathew et al. underlined that botulinum toxin may be a good solution for the patients in whom oral medications (nonsteroidal anti-inflammatory drugs, local anesthetics and gabapentin) have not been effective [46]. A 5-year observation of 1347 patients treated due to chronic headache using 100 mice units (MU) of botulinum toxin performed by Farinelli et al. has showed that the drug is effective and well tolerated by the patients. Absence of positive treatment outcomes were observed in only 1.6 % of the patients [47]. The study of Christidis et al. showed that other injection therapy is effective in muscle-related headaches e.g. repeated intramuscular tender-point injections with the serotonin type 3 antagonist granisetron are useful in myofascial temporomandibular disorders management [48]. It’s mean that in close future physicians will be able to choose treatment option from many of injection therapies concerning temporomandibular disorders-related muscle pain.

Taking into account a positive results of the BTXA injections applied in the study, it should be noted that the dose of 21 U and used operative technique are sufficient to decrease the masseter muscle pain in patients with temporomandibular joint dysfunction and tension-type headache. However we don’t know what happens after 6 months observations. We can assume that with the passage of time the effect of the neurotoxin goes away and the patient will begin to feel the pain again. We are able to repeat whole procedure in a case of recurrence. We have to know that current studies proofed that BTXA injections cause mandible bone loss and uncontrolled structural changes in affected and unaffected muscles [49, 50]. Therefore we have to emphasized that BTXA injections should be taken under consideration as a treatment of choice but not primary option in masseter muscle pain management and the dose should be kept as small as possible.

## Conclusion

The intramuscular botulinum toxin type A injections have been an efficient method of treatment in a case of masseter muscle pain in patients with temporomandibular joint dysfunction and tension-type headache. The authors recommend this therapy as a method of choice in a masseter muscle pain management.

## Abbreviations

TMJD: temporomandibular joint dysfunction
TTH: tension-type headache
BTXA: botulinum toxin type A
VAS: visual analogue scale
VNRS: verbal numerical rating scale
